# Polygenic discrimination of leaf morphotypes in alpine *Heldreichia bupleurifolia* (Brassicaceae) across its natural range

**DOI:** 10.1093/aob/mcaf223

**Published:** 2025-09-12

**Authors:** Emrullah Yılmaz, Ilgın Deniz Can, Kurtuluş Özgişi, Hakan Gür, İsmail Kudret Sağlam, Barış Özüdoğru

**Affiliations:** Department of Biology, Faculty of Science, Eskisehir Technical University, Eskişehir, Türkiye; Department of Biology, Hacettepe University, Ankara, Beytepe 06800, Türkiye; Department of Biology, Eskişehir Osmangazi University, Eskişehir, Büyükdere 26040, Türkiye; Anatolian Biogeography Research Laboratory, Kırşehir Ahi Evran University, Kırşehir, Türkiye; Department of Molecular Biology and Genetics, Koç University, İstanbul, Rumelifeneri 34450, Türkiye; Department of Biology, Hacettepe University, Ankara, Beytepe 06800, Türkiye

**Keywords:** Alpine plants, Brassicaceae, Gene Ontology (GO), polygenic traits, population genomics

## Abstract

**Background and Aims:**

Leaf morphology is a critical adaptive trait in plants, yet its genetic basis and environmental drivers remain poorly resolved in natural populations. *Heldreichia bupleurifolia*, a polymorphic alpine species in Turkey, exhibits three distinct leaf morphotypes (entire, mixed, lobed/dissected) across a west-to-east geographical gradient. This study aims to investigate genome-wide patterns of genetic variation associated with these morphotypes, identify candidate loci and pathways involved in phenotypic differentiation, and examine potential environmental correlates/drivers.

**Methods:**

Using RADseq, we generated 61 286 high-confidence single nucleotide polymorphisms from 131 individuals across 20 populations. Genetic structure was analysed via principal components analysis, while morphotype differentiation was assessed using discriminant analysis of principal components (DAPC) and random forest (RF) classification. Gene Ontology enrichment analysis identified biological pathways linked to candidate loci. Field observations correlated morphotypes with substrate type.

**Results:**

Three genetic clusters aligned with a west-to-east geographical gradient, with limited congruence to traditional subspecies classifications. DAPC and RF achieved perfect discrimination among morphotypes, identifying 167 loci (90 from DAPC, 83 from RF; five shared) localized to protein-coding regions. Enriched pathways included ion homeostasis and transmembrane transport, critical for nutrient-poor alpine soils. Lobed/dissected-leaved populations exclusively occupied volcanic substrates, while entire/mixed-leaved groups occurred on limestone, highlighting substrate-driven selection.

**Conclusion:**

Our findings suggest that genetic differentiation among morphotypes may involve stress-related processes, particularly ion transport and homeostasis. The enrichment of these functions among candidate loci, alongside observed correlations between morphotypes and substrate types, also hints at a possible contribution of edaphic conditions. While these associations are correlative and require further validation, they underscore potential links between genotype, phenotype and environment, and highlight the value of integrating genomic and ecological data in alpine plant conservation.

## INTRODUCTION

Plants, as sessile organisms, are directly exposed to environmental challenges, making adaptation to local conditions vital. Leaves, as the main photosynthetic organs in most plants, are essential for plant fitness. Their shape and dissection patterns play a critical role in optimizing light absorption and regulating gas, water and heat exchange with the environment. Therefore, they vary significantly in shape to adapt to specific environmental challenges (Adams *et al.*, 2018; Ferris, 2019). For example, the degree of leaf dissection, or the extent to which a leaf is divided into lobes or leaflets, is known to be highly associated with environmental gradients. More dissected leaves can maintain higher rates of photosynthesis, especially during hot and dry conditions, by reducing water stress through improved heat transfer (Ramirez *et al.*, 2020 ). Additionally, leaf dissection is negatively correlated with hydraulic resistance, potentially allowing plants to better cope with drought or arid environmental conditions (Gurevitch, 1988; Ramirez *et al.*, 2020). The degree of leaf dissection has been observed to vary along environmental gradients, such as altitude, suggesting its importance in local adaptation (Gurevitch, 1988; Maccagni and Willi, 2022).

Leaf shape, dissection pattern and size are determined by a complex interplay of genetic, developmental and environmental factors (Fritz *et al.*, 2018; Bhatia *et al.*, 2021). Recent research has focused on unravelling the genetic architecture of individual leaf traits, including the genetic basis of leaf dissection. This trait is regulated by a network of developmental genes, regulatory pathways and signalling molecules [e.g. KNOX, CUC, ARP and TCP gene families; auxin signalling; and the PIN1 transport protein (Nikolov *et al.*, 2019)]. Variations in *cis*-regulatory elements and structural changes, such as gene deletions, also contribute to the genetic architecture of leaf dissection (Sicard *et al.*, 2014).

However, most insights into the genetic basis of leaf traits come from model organisms. As a result, the relative contributions of genetic determination versus phenotypic plasticity to leaf shape variation remain poorly understood, particularly in natural populations and species adapted to diverse, dynamic environments (Royer *et al.*, 2009; Ferris *et al.*, 2015). Furthermore, the molecular mechanisms driving leaf shape diversity across plant lineages and their evolutionary implications require further investigation (Nakayama *et al.*, 2022).

In natural populations, the genetic architecture of leaf traits is probably shaped by complex gene-by-environment interactions, complicating efforts to disentangle the contributions of individual genetic loci (Chitwood and Sinha, 2016; Gupta *et al.*, 2020; Napier *et al.*, 2023). This gap in knowledge limits our ability to predict how leaf traits evolve and adapt to environmental changes, such as climate change or habitat fragmentation. Addressing these challenges is critical for understanding the genetic basis of morphological evolution in plants and improving predictions of plant responses to environmental changes. Future research should prioritize studying the genetic mechanisms underlying leaf shape and size in natural populations to bridge these knowledge gaps.

**Fig. 1.**
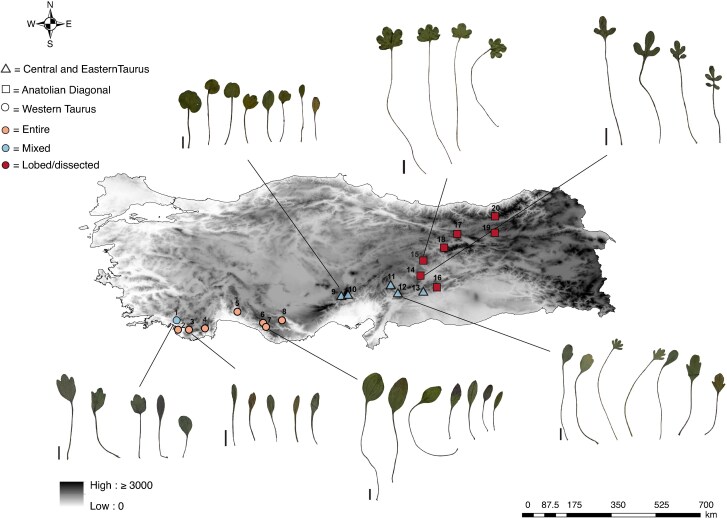
Geographical distribution and morphological classification of the 20 *Heldreichia bupleurifolia* populations. The samples were organized into geographical groups (Western Taurus Mountains, Central and Eastern Taurus Mountains, Anatolian Diagonal Mountains) and three morphological clusters: entire, mixed and lobed/dissected leaves. The names of the numbered populations are as follows: 1 = TUZLABELI, 2 = BABADAG, 3 = AN_AKDAG, 4 = ALAKIR, 5 = AKPINAR, 6 = GOKBEL, 7 = GOKBEL_2, 8 = OYUKLU, 9 = K_MANGIRCI, 10 = KARAYALAK, 11 = BERIT, 12 = ENGIZEK, 13 = M_AKDAG, 14 = LEVENT, 15 = YAMADAG, 16 **=** AD_AKDAG, 17 = KARADAG, 18 = ILIC, 19 = BAHTLI, 20 = ERENLER. For detailed population information, see Supplementary Data Table S1.

*Heldreichia* Boiss., a genus within the tribe Biscutelleae (Brassicaceae), is diploid despite the tribe’s recent history of genome duplication via autopolyploidy. This contrasts with the clear allopolyploid origins and hybrid genome architectures observed in related genera such as *Biscutella* L., *Lunaria* Tourn. ex L. and *Ricotia* L. (Kiefer *et al.* 2014; Özüdoğru *et al.*, 2017; Guo *et al.*, 2021; Hendriks *et al.*, 2023)*. Heldreichia bupleurifolia* Boiss. is a polymorphic species adapted to alpine scree habitats in the Taurus and Anatolian Diagonal Mountains of Turkey, with a geographically isolated and disjunct population also documented in Lebanon (Parolly *et al.*, 2010; Özüdoğru and Yıldırım, 2019) (Fig. 1). This species exhibits a remarkable range of leaf shapes, from simple spatulate forms to highly dissected bipinnatifid types, occurring both within and across populations (Parolly *et al.*, 2010). *Heldreichia bupleurifolia* is currently recognized to comprise six accepted intraspecific taxa: *H. bupleurifolia* subsp. *bupleurifolia*, *H. bupleurifolia* subsp. *bourgaei* (Boiss.) Parolly, Nordt & Mumm., *H. bupleurifolia* subsp. *malatyana* Özüdoğru & Yıldırım, *H. bupleurifolia* subsp. *polymorpha* Parolly, Nordt, Eren & Mumm., *H. bupleurifolia* subsp. *rotundifolia* (Boiss.) Parolly, Nordt & Mumm. var. *rotundifolia* and *H. bupleurifolia* subsp. *rotundifolia* (Boiss.) Parolly, Nordt & Mumm. var. *atalayi* (Kit Tan) Parolly, Nordt & Mumm. Subspecies designations have traditionally been based on variations in basal leaf morphology. However, both published studies and extensive field research reveal that these subspecies classifications do not align with the observed distribution of leaf shapes across the species range. Both simple and lobed leaves, once thought to distinguish subspecies, often coexist on the same individual, particularly in certain populations (Parolly *et al.*, 2010; Özüdoğru and Yıldırım, 2019). For example, the mixed leaves attributed to *H. bupleurifolia* subsp. *polymorpha* – initially described from the westernmost distribution – are also observed throughout the central–eastern range of the species (Parolly *et al.*, 2010) (Fig. 1).

Regardless of subspecies designation, three distinct basal leaf morphotypes can be identified along a west-to-east geographical gradient: (1) populations with entire leaves, predominantly in the Western Taurus Mountains; (2) populations with mixed leaves, forming the central distribution across the central Taurus range; and (3) populations with lobed or dissected leaves, exhibiting various dissection patterns (e.g. pinnate, bipinnate) and extending from the Anatolian Diagonal Mountains into northeastern Turkey (Fig. 1).

Given this variation, *H. bupleurifolia* provides an exceptional model for investigating the genetic basis of leaf morphology across a geographical gradient and identifying loci associated with genetic discrimination of leaf morphotypes in natural populations. Therefore, the primary aim of this study was to investigate the fine-scale genetic structure and variation of *H. bupleurifolia* across its range in Turkey and to identify genomic regions associated with discrimination of morphotypes along the observed geographical gradient. By utilizing genome-wide data, we aimed to characterize patterns of genetic variation associated with leaf shape/dissection and to identify candidate regions and genes potentially involved in morphotype differentiation. These findings will offer valuable insights into the genetic architecture of leaf morphology within a natural population across its distributional range.

## MATERIALS AND METHODS

### Sampling and sequencing

Leaf samples from 136 individuals of *H. bupleurifolia* were collected across 20 different locations throughout its distribution range in Turkey and classified into three morphotypes (entire, mixed and lobed/dissected) according to leaf morphology (Supplementary Data Table S1; Fig. 1). Silica-dried leaf samples were ground with sea sand and DNA was extracted using the DNeasy Plant Mini Kit (Qiagen) following the manufacturer’s protocol, with minor adjustments to the incubation time (30 vs. 10 min) and final volume (60 vs. 200 μL). Paired-end RAD libraries were prepared using the SbfI restriction enzyme and sequenced at 10× on the Illumina platform. Library preparation and sequencing were carried out by Floragenex (Eugene, OR, USA).

### Phenotyping

This study classifies a leaf as lobed or dissected when the lamina shows marginal incisions (sinuses) extending towards the midrib or base. These incisions can vary from shallow to deep, approaching the midrib without fully dividing the blade, leading to projections that may or may not be entirely separated into leaflets. According to this classification, populations are divided into three groups: (1) those exhibiting exclusively entire (non-lobed) leaves, (2) those displaying lobed or dissected (fragmented) leaves, and (3) those with both entire and lobed leaves in the same individual. The same individuals used for phenotypic analyses were also selected for genotyping. For conservation reasons, two or three individuals from each population were made into herbarium specimens, while the morphological classification of the remaining individuals relied on field observations.

### Mapping and filtering

Individual sequences were aligned to the reference genomes of *Arabidopsis thaliana* (L.) Heynh. and *Megadenia pygmaea* Maxim. – the latter being more closely related to *Heldreichia* – using the BWA-MEM algorithm (Li, 2013) as described by Lamesch *et al.* (2012) (Yang *et al.*, 2021). Although alignment to *M. pygmaea* yielded a slightly higher success rate (69.5 % vs. 60.5 %), the gain was modest (9 %). Given the more comprehensive annotation and higher-quality assembly of the *A. thaliana* genome, we opted to proceed with it as the reference, prioritizing downstream interpretability of gene functions and pathway analyses. Alignment files in SAM (Sequence Alignment Map) format were converted to BAM (Binary Alignment Map) format, followed by sorting and indexing of aligned reads using Samtools v.1.21 (Li *et al.*, 2009). Duplicate reads were identified and marked using MarkDuplicates in Picard v.2.27.4 (Broad Institute, 2024).

### Variant discovery and genotyping

Variant discovery and genotype calling were conducted using the probabilistic framework in ANGSD (Analysis of Next Generation Sequencing Data) (Korneliussen *et al.*, 2014). Genotype likelihoods (-GL 1), genotype probabilities (-doGlf 2), major and minor alleles (-doMajorMinor 1), minor allele frequencies (-doMaf 1) and read counts (-doCounts 1) were calculated using a minimum base quality of 20 (-minQ 20) and a minimum mapping quality of 30 (-minMapQ 30). Polymorphic loci were defined using a minor allele frequency (MAF) threshold of 0.05 and a significance cutoff of 2 × 10⁻⁶ (-SNP_pval 2e-06). Sites were retained only if they exhibited an average read depth of at least 6× per individual and were present in ≥50 % of individuals within each population. Genotypes were called (-doGeno 5) using a flat prior (-doPost 2) and a posterior probability cutoff of 95 % (-postCutoff 0.95) and outputted as VCF files (-doBcf 1).

### Genetic structure

Principal component analysis (PCA) was performed using the snpR package (v.1.2.79; Hemstrom and Jones, 2023) to assess population genetic structure, employing the smartPCA algorithm (Patterson *et al.*, 2006; Price *et al.*, 2006). The first two principal components (PC1 and PC2) were plotted in R using ggplot2 (Wickham, 2016) to visualize genetic variation.

Furthermore, an admixture analysis was conducted using NGSadmix (Skotte *et al.*, 2013) to estimate individual ancestry proportions based on genotype likelihoods. We tested values of *K* ranging from 1 to 6, performing six independent runs per *K* to account for potential stochastic variation and ensure convergence. The optimal number of genetic clusters was inferred using the Δ*K* method (Evanno *et al.,* 2005), which evaluates the rate of change in log-likelihood values between successive *K* values. Admixture proportions for each *K* were visualized in R.

### Genetic discrimination of leaf morphotypes

To evaluate the extent of genetic differentiation among leaf morphotypes (entire, lobed/dissected and mixed), we employed two complementary classification approaches: discriminant analysis of principal components (DAPC) and random forest (RF).

DAPC was performed using the adegenet R package (v.2.1.1; Jombart, 2008; Jombart and Ahmed, 2011), with morphotype categories defined *a priori*. Principal components (PCs) explaining up to 80 % of cumulative genetic variance were retained, and classification performance was assessed via posterior assignment probabilities along the first discriminant function (DF1). To identify single nucleotide polymorphisms (SNPs) contributing to morphotype differentiation, locus loadings on DF1 were clustered using the snpzip function (average linkage), and loci exceeding the 99th percentile of the null distribution were retained. Genotypic variation across these significant SNPs was visualized with heatmaps generated using the pheatmap R package (Kolde, 2012), based on hierarchical clustering of genotype calls. The closest *Arabidopsis* protein-coding genes (exonic or intronic) were annotated using The Arabidopsis Information Resource (TAIR; Rhee *et al.*, 2003).

To complement the DAPC results, we performed an RF analysis using the ranger package (Wright and Ziegler, 2017) in R. Morphotype categories were used as the response variable and SNP genotypes as predictors. The model was trained using 5000 trees (ntree = 5000) and 40 variables randomly sampled at each split (mtry = 40). SNPs with missing data were removed, resulting in 1651 high-quality loci. Feature importance scores were calculated based on the Gini impurity index (a measure of each SNP’s contribution to reducing classification error), and SNPs above the median importance threshold were retained to reduce noise.

To identify the most informative subset of loci, additional RF models were trained on successively filtered SNP sets, created by applying variable importance thresholds at multiple percentile levels. Each model’s classification performance was evaluated using out-of-bag (OOB) error estimates. A backward purging procedure was then applied to iteratively remove low-ranking SNPs, with OOB error recalculated at each step to identify the most stable and predictive marker panel. The final SNP set was used for downstream visualization and gene annotation, as described above.

### Gene Ontology

Gene Ontology (GO) enrichment analysis was conducted to identify biological processes associated with SNPs identified in both DAPC and RF analyses. Candidate SNPs were converted to BED format and mapped to *A. thaliana* gene annotations (TAIR10) using bedtools intersect v.2.30.0 (Quinlan and Hall, 2010) with default parameters. Genes overlapping or adjacent to significant SNPs (±1 kb) were extracted for analysis.

To account for the reduced-representation nature of RAD sequencing (RADseq) data, we restricted the background gene set to only those *A. thaliana* genes that had at least one RAD locus successfully aligned from our dataset. This approach ensures that enrichment is evaluated relative to the genomic regions actually sampled, minimizing bias introduced by comparing against genes that could not have been detected.

Enrichment testing was performed in R using the clusterProfiler package v.4.0 (Yu *et al.*, 2012) with the org.At.tair.db annotation database (Carlson, 2019). Overrepresentation of Biological Process (BP) terms was evaluated using a hypergeometric test, and *P*-values were adjusted for multiple testing using the Benjamini–Hochberg false discovery rate (FDR) correction. GO terms with FDR < 0.2 and raw *P*-value <0.05 were considered significantly enriched. For genes lacking annotations in org.At.tair.db, additional GO term information was manually retrieved using the QuickGO database (Huntley *et al.*, 2009).

Results were visualized using bar plots (ranked by adjusted *P*-value) and cnet plots (network representations of gene–term associations), generated with enrichplot v.1.26.1 (Xu *et al.*, 2024), to highlight key pathways and functional relationships.

## RESULTS

Sequenced reads mapped to the *A. thaliana* reference genome with moderate to high efficiency (60.5 % mean alignment rate), yielding an average of 1.5 million mapped reads per individual (Supplementary Data Table S2). Based on alignment depth, five individuals from the Akpınar and Bahtlı populations with fewer than 1 000 000 mapped reads were excluded, resulting in a final dataset of 131 individuals (42 entire, 28 mixed, 61 lobed/dissected) across 20 populations. Initial variant calling identified 2 852 005 polymorphic sites (SNP *P* < 2 × 10⁻⁶). After stringent filtering (minimum read depth of 6× per individual, 50 % site occupancy per population), 61 286 high-confidence SNPs were retained. This curated dataset formed the basis for all downstream analyses and provided a high-quality genomic resource for exploring genetic structure and morphotype differentiation in *H. bupleurifolia*.

### Genetic structure

**Fig. 2.**
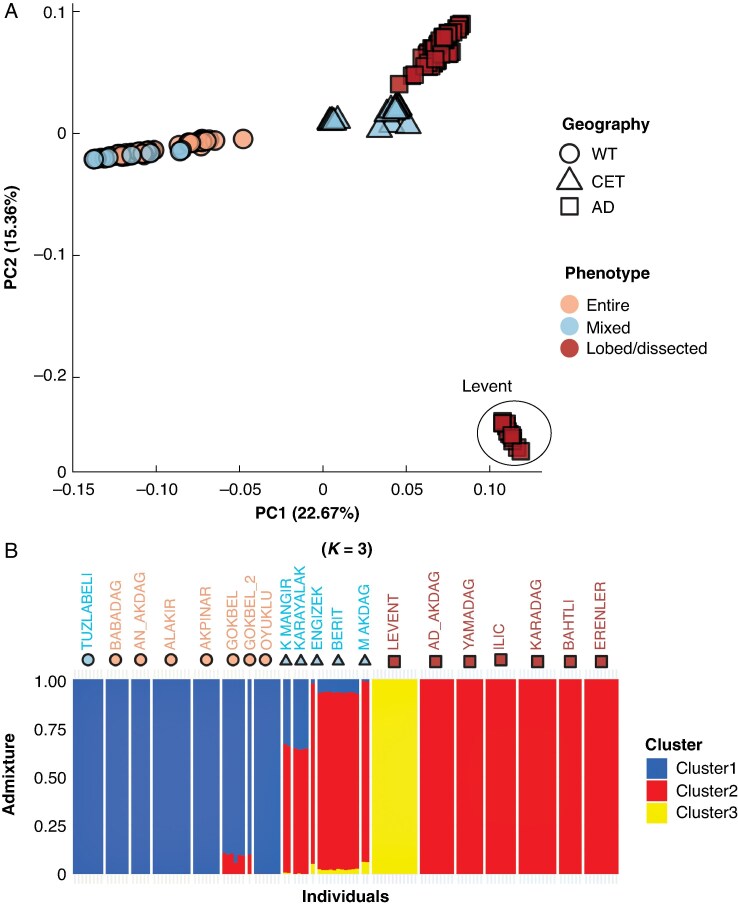
Genetic structure analyses of 20 *H. bupleurifolia* populations. (A) Principal component analysis (PCA) of genetic variation for *Heldreichia bupleurifolia* based on 61 286 SNPs. Dots, triangles and squares indicate the geographical location of the populations. WT = Western Taurus, CET = Central and Eastern Taurus, AD = Anatolian Diagonal. Main leaf morphotypes are indicated by colour. In the diagram, all the blue circles positioned among the populations with entire leaves (apricot-coloured circles) represent individuals from the TUZLABELI population. (B) NGS admixture plot with *K* = 3. The colours of the population names match the phenotype colours in the PCA diagram.

PCA revealed strong geographical structuring of genetic variation in *H. bupleurifolia* populations (Fig. 2A). The first two principal components (PC1 and PC2) explained 22.67 and 15.38 % of the total genetic variance, respectively. Populations were separated along the PC1 axis, with a clear west-to-east cline reflecting spatial proximity rather than leaf morphotype. Individuals from the same geographical region – including those with distinct leaf morphotypes (entire, mixed, lobed/dissected) – clustered together, indicating shared genetic structure within localities. Notably, the westernmost population (*H. bupleurifolia* subsp. *polymorpha*; Fethiye–Tuzlabeli, Supplementary Data Table S1), characterized by mixed-leaf phenotypes, clustered genetically with nearby populations dominated by entire-leaf morphotypes, further supporting spatial proximity as the primary driver of overall genome-wide genetic divergence.

ADMIXTURE analysis identified *K* = 3 as having the highest Δ*K* value, suggesting that the most supported genetic structure consists of three well-defined clusters (Fig. 2B; Fig. S1). These clusters correspond to populations distributed across the western Taurus, central and eastern Taurus plus Anatolian Diagonal (AD) and one population (Levent) from AD corresponding to subsp. *malatyana* (Fig. 2B). Among the mixed-leaved populations, those from the central and eastern Taurus exhibit a clear signature of genetic admixture between Cluster 1 (blue) and Cluster 2 (red). In contrast, the mixed-leaved population from Tuzlabeli is consistently and predominantly assigned to Cluster 1 (blue), suggesting a homogeneous and regionally consistent genetic background with adjacent populations (Fig. 2B), as also evident in the PCA.

### Genetic differentiation based on leaf morphology

**Fig. 3.**
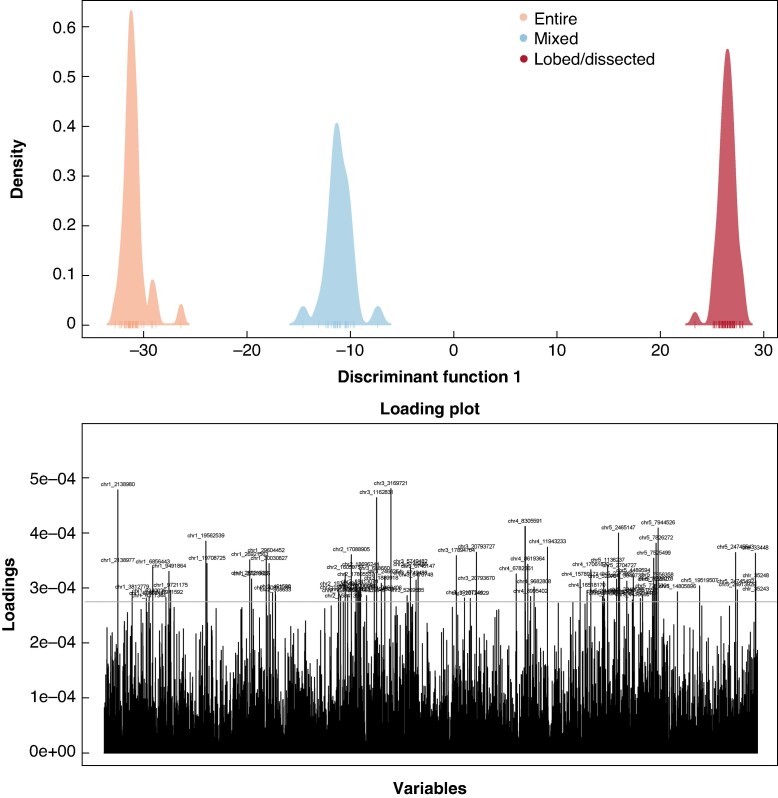
Discriminant analysis of principal components (DAPC) plots illustrating the variation among 131 *Heldreichia bupleurifolia* samples genotyped across 61 286 SNPs. (A) Density of *H. bupleurifolia* individuals along the discriminant function generated by DAPC. (B) Loading plot highlighting how SNPs contribute to the DAPC. The average clustering method was applied to the distance matrix to classify loci into those influencing group divergence and those without a significant effect. A black horizontal line marks the threshold.

**Fig. 4.**
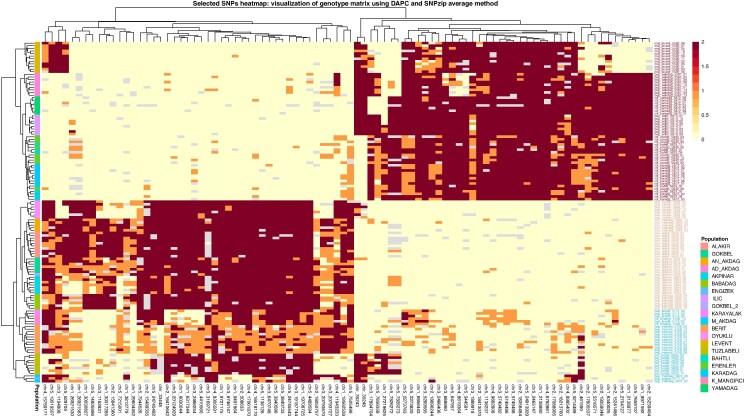
Sample location allele frequencies for the 90 most important SNPs based on DAPC results to distinguish *Heldreichia bupleurifolia* leaf morphotypes. Each column represents a specific SNP, and each row represents an individual used in this study. The colour code used for the individuals corresponds to the colours in Fig. 1.

**Fig. 5.**
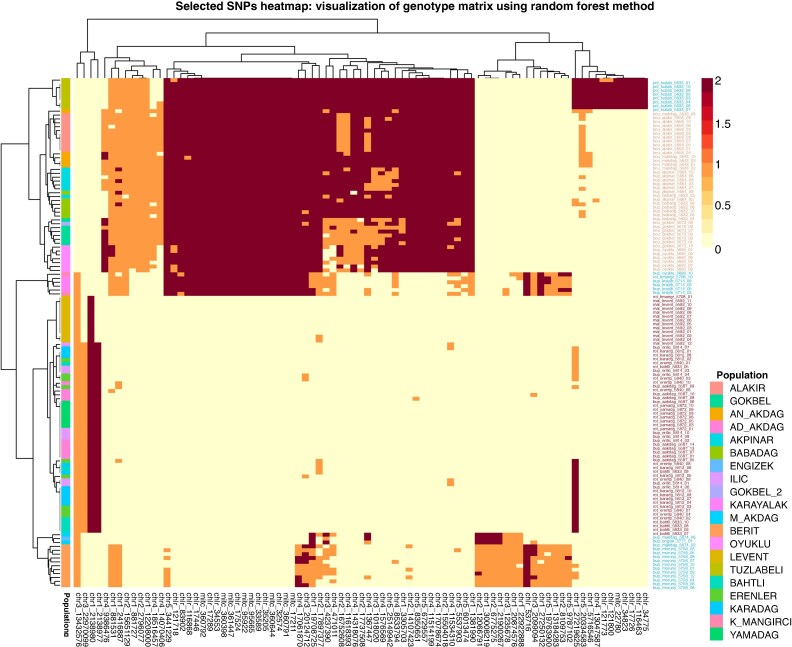
Sample location allele frequencies for the 83 most important SNPs based on random forest results to distinguish *Heldreichia bupleurifolia* leaf morphotypes. Each column represents a specific SNP, and each row represents an individual used in this study. The colour code used for the individuals corresponds to the colours in Fig. 1.

Both DAPC and RF classification provided clear evidence of genetic differentiation among the three leaf morphotypes (entire, mixed and lobed/dissected). DAPC achieved perfect discrimination along the first discriminant function (DF1), with posterior assignment probabilities of 1.0 for all groups (Fig. 3A). Hierarchical clustering of SNP contributions to DF1 (snpzip; average method) identified 90 significant loci exceeding the 99th percentile of the null distribution (Fig. 3B). These loci showed substantial genotype partitioning by morphotype, particularly between lobed/dissected and the other two morphotypes (entire and mixed) (Figs 4 and 5). Furthermore, all 90 SNPs were located within protein-coding genes (exonic or intronic regions) (Supplementary Data Table S3).

RF classification corroborated the DAPC results and further highlighted a compact set of highly informative SNPs. After filtering and iterative model optimization, a final panel of 83 SNPs yielded the lowest OOB error and achieved 100 % classification accuracy across morphotype categories. These SNPs, like those identified in the DAPC, showed clear genotype structuring among leaf types and were all located within or near annotated protein-coding genes in *A. thaliana*. The convergence of both approaches on strongly discriminating SNP sets underscores the robust genetic signal underlying leaf shape differentiation in *H. bupleurifolia*.

### Gene Ontology

**Fig. 6.**
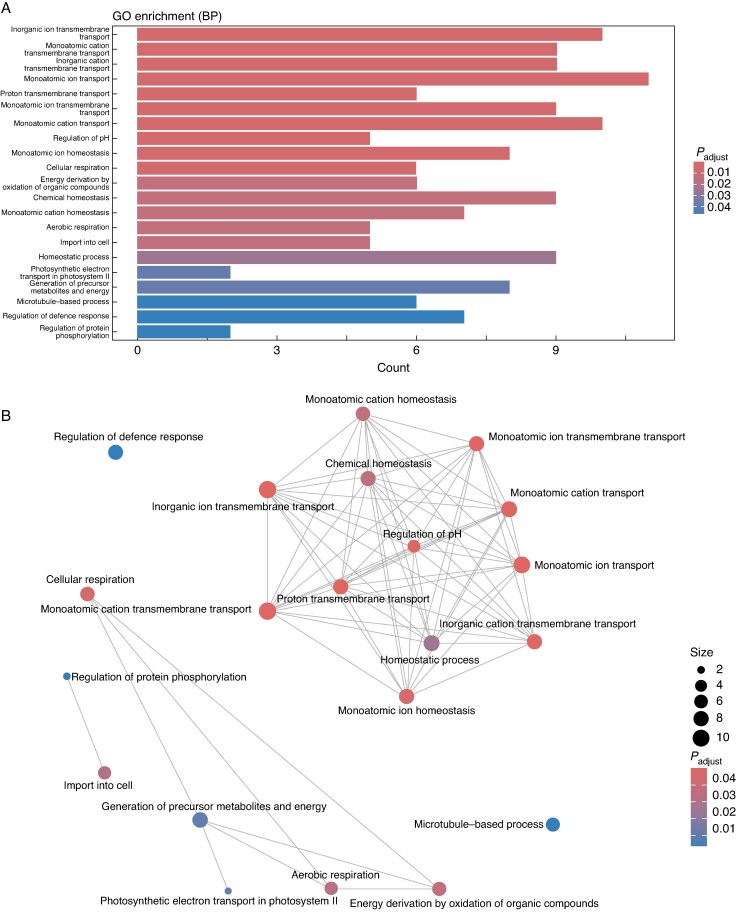
Significantly enriched biological processes identified through GO enrichment analysis of SNP-associated genes. Bars represent the count of genes per term, with colours indicating adjusted *P*-values.

Of the 90 and 83 SNPs identified in the DAPC and RF analyses, respectively, five were shared between the two (Supplementary Data Table S3). This yielded 167 loci, which were annotated by mapping SNP coordinates ±1 kb to gene models in the TAIR10 reference genome. A total of 137 protein-coding genes were found to overlap with these SNPs, located primarily within exonic or internal intronic regions. These genes were used in a GO enrichment analysis, which revealed 21 significantly enriched biological processes (FDR < 0.2; *P* < 0.05; Fig. 6), including transport and homeostasis pathways relevant to phenotypic differentiation and development.

## DISCUSSION

The genomic resources generated here – 61 286 high-confidence SNPs derived from RADseq – provide the first comprehensive genome-wide assessment of genetic diversity and population structure in *H. bupleurifolia* across its natural range. This represents a significant advance over earlier studies from the Anatolian Mountains, which largely relied on one or two loci (e.g. ITS, chloroplast markers) to infer genetic patterns (Ansell *et al.*, 2011; Koch *et al.*, 2017; Özüdoğru *et al.*, 2022 ). Our genome-wide approach resolves fine-scale population structure and identifies candidate loci that may play a role in leaf morphotype differentiation. Considering the strong correlation among leaf morphology, substrate type and genome-wide structure, some associations may reflect broader spatial or ecological divergence rather than direct trait-specific selection. Nonetheless, our findings offer a critical step toward in understanding how polygenic traits evolve and become structured in complex natural systems.

### Population structure challenges subspecies classifications

Traditional subspecies classifications of *H. bupleurifolia*, based on basal leaf morphology (Parolly *et al.*, 2010), do not align with the genetic clusters uncovered here. Genome-wide analyses reveal a clear west-to-east gradient, forming three primary clusters: Western Taurus (WT), Central and Eastern Taurus (CET) and Anatolian Diagonal (AD) (Fig. 2). The boundaries between subspecies largely break down within the WT and CET clusters, as individuals from multiple subspecies are intermixed and do not form clearly separated groups in either PCA or admixture analyses. In contrast, the AD cluster comprises two distinct groups in PCA that correspond to subsp. *malatyana* and subsp. *rotundifolia*, respectively. This separation is further supported by admixture analysis, which assigns these two subspecies to different genetic clusters and reveals little admixture between them. These findings emphasize how phenotypic plasticity or convergent evolution – both capable of producing similar leaf morphologies in genetically distinct populations – can obscure taxonomic boundaries, a well-documented challenge in plant systematics (Royer *et al.*, 2009; Ferris *et al.*, 2015), and underscore the importance of disentangling neutral demographic processes from adaptive divergence when making taxonomic inferences.

A striking example is the Tuzlabeli population (WT, subsp. *polymorpha*), which exhibits mixed leaf forms. While the PCA, which reflects patterns of genome-wide (largely neutral) genetic variation, clusters the Tuzlabeli population with other Western Taurus entire-leaved populations, both DAPC and RF analyses – focused specifically on phenotype-associated genetic differentiation – assign it to the mixed-leaved group with 100 % accuracy. This discrepancy between genotype and phenotype underscores the need to critically examine morphotype classifications, especially in the context of local adaptation or plasticity.

### Genetic basis and architecture of leaf morphology

Both DAPC and RF analyses achieve perfect discrimination among leaf morphotypes, implicating a polygenic architecture for this trait. The 90 and 83 loci identified, respectively, by these approaches were exclusively located in protein-coding regions, with five shared between them (Supplementary Data Table S2). This convergence supports the robustness of genotype–phenotype associations and suggests selection on coding variation.

Interestingly, while leaf morphology is typically associated with canonical developmental regulators – such as those involved in auxin signalling (Nikolov *et al.*, 2019) – GO enrichment analysis revealed a predominant enrichment of ion homeostasis-related pathways, alongside smaller clusters related to transmembrane transport (FDR < 0.2; Fig. 6). Only three genes (AT2G28290, AT4G03350, AT4G16670) were strong candidates for direct roles in leaf dissection. Among these, AT2G28290 (SYD) modulates CUC2, a regulator of organ boundaries and leaf edge patterning (Kwon *et al.*, 2006); AT4G03350 (GSL05) contributes to leaf morphogenesis; and AT4G16670 (DUF828) affects vascular patterning (Hou *et al.*, 2010; Hwang *et al.*, 2011).

### Environmental context and substrate associations

Our genomic analysis identified significant enrichment of gene pathways related to monoatomic ion transport, cation transmembrane transport and ion homeostasis, based on loci most strongly associated with leaf morphotype variation. These pathways are critical for maintaining cellular ion balance, nutrient uptake and turgor regulation – key functions in plant stress physiology (Assaha *et al.*, 2017). Although our enrichment analysis was focused specifically on genetic associations with leaf shape, it is notable that similar pathways have been implicated in plant responses to edaphic stressors in alpine environments (Mulet *et al.*, 2023; Mishra *et al.*, 2024), suggesting that genes linked to leaf morphology may also have roles in substrate-related adaptation. This interpretation is further supported by the ecology of *H. bupleurifolia*, which is adapted to scree habitats in the Taurus and Anatolian Diagonal Mountains – environments marked by low nutrient availability, high UV and temperature extremes (Parolly *et al.*, 2010; Hou *et al.*, 2024).

Genotype-based heatmaps further reveal that lobed/dissected-leaf morphotypes are genetically distinct whereas populations with entire or mixed leaves show less clear genetic separation (Figs 4 and 5). Field observations add another layer to this pattern: lobed/dissected populations are found exclusively on volcanic and plutonic substrates, while entire and mixed-leaved populations occur on limestone bedrock. While this substrate–morphotype association is compelling, we emphasize that it is correlational and does not establish causation. Some preliminary evidence from controlled growth conditions further suggests a genetic basis for leaf dissection that is independent of immediate growth conditions (i.e. immediate environmental effects). In small-scale trials, individuals retained their parental morphotypes when grown under standardized conditions (Supplementary Data Fig. S2) Although limited in scope, these observations support the hypothesis that leaf morphology has a heritable component, and may reflect long-term local adaptation rather than phenotypic plasticity.

However, the spatial alignment of genetic differentiation, leaf morphology and substrate type raises the possibility that edaphic conditions may contribute to morphotype divergence – either through direct selection or as part of broader environmental gradients. Previous studies in alpine systems have documented similar links between soil chemistry and morphotype distribution (Gurevitch, 1988; Ramirez *et al.*, 2020), lending support to this hypothesis.

These overlapping axes of morphological, ecological and genetic variation make it challenging to fully disentangle the causes of observed genotype–phenotype associations. It remains possible that some loci identified as candidates for leaf shape differentiation, particularly those involved in ion transport, stress response or cytoskeletal remodelling, may be indirectly associated with morphology due to underlying correlations with edaphic adaptation. While pleiotropic functions are plausible for some genes, others may reflect parallel responses to shared environmental gradients rather than direct morphological control. We therefore interpret our candidate loci as indicative of potential pathways linked to morphotype divergence, either causally, pleiotropically or through environmental covariance.

Moreover, several microtubule-related GO terms (e.g. *AT1G18410*, *AT1G19835*, *AT5G08390*) were also enriched among candidate loci. In *Arabidopsis*, microtubule reorganization (e.g. via KATANIN-mediated severing) plays a key role in stress response and cell wall remodelling under mechanical pressure (Burk *et al.*, 2001; Sampathkumar *et al.*, 2014; Bidhendi *et al.*, 2019). Given that *Heldreichia* populations occur on unstable, erosion-prone substrates, such genes may contribute to morphological adaptation under mechanical or substrate-induced stress.

Together, our findings suggest that the genetic architecture underlying leaf morphology may intersect with pathways relevant to stress tolerance and environmental responsiveness. While further functional validation is needed, the observed overlap between morphotype-associated loci and stress-relevant pathways highlights a potential link between leaf shape, genotype and habitat.

### Limitations and future directions

Our failure to recover canonical developmental genes may reflect both biological and methodological limitations. First, RADseq interrogates only ∼1–2 % of the genome, often missing regulatory elements and low-frequency alleles involved in complex traits. Second, while genetic regulators of leaf development are well characterized in model organisms (Nikolov *et al.*, 2019), their modulation by environmental factors in non-model alpine systems remains poorly understood.

Indeed, RADseq’s limited and non-random genomic scope (Davey *et al.*, 2011; Lowry *et al.*, 2017) introduces a conservative bias: associations identified are probably robust, but many relevant loci may be missed entirely. This necessitates caution when interpreting negative results (Hoban *et al.*, 2016). Yet, the strong genotype–phenotype signals observed here, despite these limitations, suggest that the identified loci reflect biologically meaningful associations. Future studies using whole-genome resequencing or targeted capture of candidate regions will be essential to comprehensively resolve the polygenic architecture of leaf morphology in *H. bupleurifolia*.

### Broader implications and conservation

Our study identified loci associated with leaf morphotype differentiation that are enriched for functions related to ion transport and cellular stress response. These gene functions are consistent with physiological processes that mediate plant responses to environmental stressors such as nutrient limitation and soil chemistry, suggesting a possible ecological relevance to morphotype-associated genetic variation. At the same time, they underscore the difficulty of fully disentangling direct trait associations from environmental and demographic correlates in structured natural systems.

While the spatial correspondence between genetic differentiation, phenotype and substrate type raises the possibility of local adaptation, the relative roles of selection, phenotypic plasticity and genetic drift remain unresolved. Further integration of soil chemistry, functional assays and common-garden experiments will be essential to disentangle these mechanisms.

Nonetheless, the strong genotype–phenotype associations revealed in this study highlight the value of investigating natural variation in complex, non-model systems. Such approaches can uncover gene–environment interactions often masked in controlled experimental settings (Gupta *et al.*, 2020; Napier *et al.*, 2023). As *H. bupleurifolia* faces mounting climatic pressures, loci involved in stress-related pathways may serve as useful biomarkers for identifying resilient genotypes. Conservation strategies should prioritize genetically diverse populations harbouring these variants to preserve evolutionary potential under future environmental change (Prince *et al.*, 2017; Thompson *et al.*, 2019).

## Supplementary Material

mcaf223_Supplementary_Data

## Data Availability

The raw sequencing reads have been submitted to the NCBI Sequence Read Archive (SRA) under accession number PRJNA1272478. All original contributions of this study have been deposited in the online repository [GitHub: github.com/emrullahub/heldreichia_paper] and are publicly available. Further inquiries should be directed to the corresponding author.

## SUPPLEMENTARY DATA

Supplementary data are available at *Annals of Botany* online and consist of the following. Table S1. Collector numbers, population names, location, geographical region codes, geographical coordinates, altitudes, collection dates, sample sizes and basal leaf morphotypes of *Heldreichia bupleurifolia*. Table S2. Population information, sequencing statistics and alignment metrics for *Heldreichia buplerifolia* samples. Table S3. Genes associated with SNPs and their functional annotations. Figure S1. Admixture analysis of *Heldreichia bupleurifolia* (*n* = 131) based on 58 777 polymorphic sites as inferred with ANGSD. Admixture diagrams are shown for *K* = 2–6. Ancestry proportions inferred with NGSadmix are shown as vertical bars, where each bar represents an individual. Figure S2. Two different morphotypes of *H. bupleurifolia* grown under the same conditions and their natural photos. (A–B) Entire and dissected-leaved samples; photos by T. Mandáková. (C–D) Natural photos.
