# Supplementary material for: Polygenic discrimination of leaf morphotypes in alpine Heldreichia bupleurifolia (Brassicaceae) across its natural range
Source: Ann Bot. 2025 Sep 12;138(3):918–29. doi: 10.1093/aob/mcaf223 (PMC13487426; doi:10.1093/aob/mcaf223)
Supplement: Supplementary file 1 [file mcaf223_supplementary_data.zip › Table_S1.docx]

Table S1. Collector numbers, population names, location, geographic region codes, geographic coordinates, altitudes, collection dates, sample sizes,basal leaf morphotypes, and bedrock types of *Heldreichia bupleurifolia.* WT= Western Taurus, CET=Central and eastern Taurus, AD=Anatolian Diagonal.

| Collector No. | Population Name | Location | Geographical regions | Latitude | Longitude | Altitude (m) | Collection Date | n | Basal leaf morphotypes | Bedrock type |
| --- | --- | --- | --- | --- | --- | --- | --- | --- | --- | --- |
| BÖ 5633 | TUZLABELI | Muğla, Fethiye-Çameli, Tuzlabeli, north facing side | WT | 36.85934 | 29.18242 | 1466 | 06/06/2021 | 8 | mixed | limestone |
| BÖ 5632 | BABADAG | Muğla, Fethiye, Babadağ | WT | 36.530568 | 29.180773 | 1940 | 06/06/2021 | 6 | entire | limestone |
| BÖ 5643 | AN_AKDAG | Antalya, Gömbe, Akdağ Mountain, upper Şemsi Village | WT | 36.50655 | 29.59821 | 2126 | 06/06/2021 | 5 | entire | limestone |
| BÖ 5658 | ALAKIR | Antalya, Kumluca, Upper Alakır | WT | 36.57745 | 30.1697 | 2200 | 16/06/2021 | 10 | entire | limestone |
| BÖ 5664 | AKPINAR | Antalya, Manavgat, upper Burunçalı, Akpınar Highland | WT | 37.14741 | 31.32223 | 1653 | 17/06/2021 | 9 | entire | limestone |
| BÖ 5673 | GOKBEL | Antalya, Alanya, Gökbel Highland Road, 5 km to Gökbel before Alacami | WT | 36.65101 | 32.32842 | 1572 | 18/06/2021 | 6 | entire | limestone |
| BÖ 5676 | GOKBEL_2 | Antalya, Alanya, beyond Gökbel Highland, the slopes facing north | WT | 36.701 | 32.2921 | 1818 | 18/06/2021 | 1 | entire | limestone |
| BÖ 5693 | OYUKLU | Karaman, Ermenek, Oyuklu Mountain | WT | 36.83303 | 32.92106 | 2087 | 19/06/2021 | 7 | entire | limestone |
| BÖ 5708 | K_MANGIRCI | Niğde, Aladağlar, Küçükmangırcı Valley | CET | 37.755313 | 35.104146 | 2060 | 23/06/2021 | 2 | mixed | limestone |
| BÖ 5714 | KARAYALAK | Niğde, Çamardı, Aladağlar, Karayalak Valley | CET | 37.80932 | 35.11995 | 2363 | 24/06/2021 | 4 | mixed | limestone |
| BÖ 5758 | BERIT | Kahramanmaraş, Berit Mountain, Morun Yatağı area | CET | 37.99918 | 36.8392 | 2461 | 26/06/2021 | 11 | mixed | limestone |
| BÖ 5777 | ENGIZEK | Kahramanmaraş, Engizek Mountain, southern slopes | CET | 37.78515 | 37.11907 | 1940 | 27/06/2021 | 1 | mixed | limestone |
| BÖ 5874 | M_AKDAG | Malatya, Erkenek, Akdağ southern slopes | CET | 37.90465 | 37.95991 | 1785 | 11/07/2021 | 2 | mixed | limestone |
| BÖ 5592 | LEVENT | Malatya, Levent Valley | AD | 38.39883 | 37.9146 | 1263 | 29/05/2021 | 12 | lobed/dissected | argillaceous carbonate rock |
| BÖ 5872 | YAMADAG | Malatya, Hekimhan, rocks beneath the Yama Mountain radar | AD | 39.06619 | 37.94066 | 2590 | 11/07/2021 | 7 | lobed/dissected | volcanic |
| BÖ 5587 | AD_AKDAG | Adıyaman, Çelikhan, southern slopes of the mountain Akdağ | AD | 37.98278 | 38.34219 | 1122 | 29/05/2021 | 9 | lobed/dissected | plutonic |
| BÖ 5812 | KARADAG | Erzincan, Karadağ Mountain | AD | 39.900165 | 39.130156 | 2630 | 06/07/2021 | 10 | lobed/dissected | plutonic |
| BÖ 5814 | ILIC | Erzincan, İliç | AD | 39.42824 | 38.61392 | 1243 | 06/07/2021 | 8 | lobed/dissected | plutonic |
| BÖ 5833 | BAHTLI | Erzurum, Kop Pass, Bahtlı Mountain | AD | 40.04085 | 40.50561 | 2558 | 08/07/2021 | 9 | lobed/dissected | argillaceous carbonate rock |
| BÖ 5840 | ERENLER | Bayburt, Erenler Hill | AD | 40.51476 | 40.51677 | 3107 | 09/07/2021 | 9 | lobed/dissected | plutonic |
